# Closing the Loop: A Re-Audit of Mental Health Tribunal Report Quality and Timeliness in a CAMHS Inpatient Setting

**DOI:** 10.1192/bjo.2026.11809

**Published:** 2026-06-30

**Authors:** Dohwodese Ohwovoriole, Amith Paramel, Ahmad Sharique, Sameer Alji-Mohamed, Lin John

**Affiliations:** Lancaster and South Cumbria NHS Foundation Trust, Preston, United Kingdom

## Abstract

**Aims:**

We set out to finish the audit loop started in 2024 at The Cove CAMHS unit. Our goal was straightforward: to prove that the fixes we put in place were actually working. Specifically, we wanted to see if tribunal reports were finally being submitted on time and if clinicians were properly documenting their conversations with the young people they treat. We also needed to ensure that our clinical standards didn’t slip during a period of systemic change, particularly with the introduction of a new digital reporting template late in the year.

**Methods:**

We compared two years of tribunal reports side-by-side: the 2024 baseline and the 2025 re-audit. In total, we reviewed every single report produced across both years (N=28) against 27 mandatory standards from the MHA Code of Practice. The interventions we tested between the two years included a new administrative tracking system for legal deadlines and a clearer clinical pathway designed to make sure patient discussions weren’t just happening, but were being recorded accurately in the notes.

**Results:**

Overall compliance climbed from 94.4% to 97.1%. Our biggest success was in timeliness: we moved from a failing 64.3% to a perfect 100% submission rate. We also saw documented patient discussions more than double, jumping from 28.6% to 64.3%. However, the data revealed a new problem: a compliance dip (85.2%) directly linked to the new digital template. Because certain prompts were missing in the new software, clinicians started omitting patient strengths and detention justifications–a critical finding that highlights the hidden risks of digital transitions.

**Conclusion:**

Completing this audit cycle confirms that administrative delays in tribunal reporting are solvable through better process mapping. While reaching 100% timeliness is a significant win for the ward, the 64.3% compliance in documenting patient discussions shows that clinical culture takes longer to change than administrative rules. Our discovery of a “transition gap” with the new template warns that digital updates can inadvertently sideline statutory details. We must now move beyond administrative fixes to focus on meaningful engagement, ensuring the “voice of the child” remains central to the tribunal process, regardless of the reporting format used.

